# 1402. Expand accessibility of monoclonal antibody in COVID-19 among different ethnicities and races.

**DOI:** 10.1093/ofid/ofac492.1231

**Published:** 2022-12-15

**Authors:** Hina Farooq, Shivanjali Shankaran

**Affiliations:** RUSH university medical center, Chicago, Illinois; RUSH university medical center, Chicago, Illinois

## Abstract

**Background:**

Anti-SARS-CoV-2 monoclonal antibodies are administered to patients with mild-moderate COVID-19 who are at high risk of progression to severe disease. It has been shown that in addition to medical comorbidities, race or ethnicity may also place patients at high risk for progression to severe COVID-19 infection due to social disparities including limited access to care. The purpose of this study is to increase the accessibility of monoclonal antibody infusion to patients at high risk for severe outcomes of COVID-19, irrespective of race and ethnicity, by expanding outreach resources when availability of oral antiviral therapies was limited.

**Methods:**

We performed a single-center retrospective analysis of patients with mild-moderate COVID-19 infection receiving sotrovimab, a monoclonal antibody, between December 2021 and January 2022. A total of 93 SARS-CoV-2-positive patients meeting EUA criteria for eligibility were infused with sotrovimab in different settings such as emergency department, outpatient setting including infusion clinics and cancer centers, home health as well as patients hospitalized due to reasons other than COVID-19 at RUSH medical center, Chicago. For context, during omicron surge, initially home health was set up followed by introduction to infusion clinics. Primary care provider could refer patients to the infusions clinics or home health.

**Results:**

Out of 93 patients, 8 patients received mAb infusion in emergency department, 25 patients each in cancer center and infusion clinic, 17 patients in home health setting and 18 patients who were hospitalized due to reasons other than COVID-19. The median age of participants was 57 years and 61.2% were females. Overall, Hispanic patients received mAb less often than did non-Hispanic patients (33% vs 62%). Black, Asian and other racial groups received mAb 18.2%, 3.23%, 3.23% less often, respectively, than did White patients. Interestingly, in home health setting, Hispanic patients received infusion more often than non-Hispanic patients (12.9% vs 5.3%).

**Conclusion:**

Implementation of programs centered around needs of community such as increase accessibility to COVID-19 medications through home health or infusion clinics may help mitigate the racial and ethnic disparities in COVID-19 and thus, promote health equity.

**Disclosures:**

**All Authors**: No reported disclosures.

